# Arterial Stiffness Predicts the Outcome of Endovascular Treatment in Patients with Acute Ischemic Stroke

**DOI:** 10.3390/jcm13144198

**Published:** 2024-07-18

**Authors:** Minho Han, Haram Joo, Hyungwoo Lee, JoonNyung Heo, Jae Wook Jung, Young Dae Kim, Eunjeong Park, Hyo Suk Nam

**Affiliations:** 1 Department of Neurology, Yonsei University College of Medicine, Seoul 03722, Republic of Korea; umsthol18@yuhs.ac (M.H.); hwlee625@yuhs.ac (H.L.); jnheo@yuhs.ac (J.H.); jaewook0730@yuhs.ac (J.W.J.); neuro05@yuhs.ac (Y.D.K.); eunjeong-park@yuhs.ac (E.P.); 2 Integrative Research Center for Cerebrovascular and Cardiovascular Diseases, Yonsei University College of Medicine, Seoul 03722, Republic of Korea

**Keywords:** arterial stiffness, endovascular treatment, ischemic stroke, outcome

## Abstract

**Background:** The association between arterial stiffness and outcome after endovascular treatment (EVT) is unknown. This study investigated whether arterial stiffness predicts post-EVT outcome in patients with acute ischemic stroke. **Methods:** This retrospective and observational cohort study included consecutive patients treated with EVT for acute ischemic stroke from June 2020 to November 2022. Arterial stiffness was assessed by brachial–ankle pulse wave velocity. Poor functional outcome was defined as a modified Rankin Scale score ≥3 at 3 months. **Results:** The mean age of patients included in this study was 71.9 ± 11.8 years, and 57.3% were men. Poor functional outcome was present in 46.8%. Multivariable logistic regression analysis showed that arterial stiffness was independently associated with poor functional outcome (odds ratio 8.640, 95% confidence interval [CI] 1.581–47.228) after adjusting for age, initial stroke severity, hypertension, atrial fibrillation, device pass number, and successful recanalization. A nomogram based on the multivariable statistic model showed a better prediction of poor functional outcome compared to classic risk factor models without arterial stiffness (net reclassification improvement 0.529, 95% CI 0.186–0.873; integrated discrimination improvement 0.046, 95% CI 0.009–0.083). **Conclusions:** We found that arterial stiffness was an independent predictor of poor functional outcome in patients treated with EVT following acute ischemic stroke.

## 1. Introduction

Endovascular treatment (EVT) has emerged as a cornerstone in the management of patients with acute ischemic stroke due to large vessel occlusion. EVT offers the possibility for significant improvements in outcomes and quality of life [1]. Pooled analyses of randomized trials have shown the consistent efficacy of EVT across diverse populations in acute ischemic stroke [2]. However, approximately half of patients with successful recanalization fail to achieve functional independence at 3 months [3]. Older age, severe stroke, longer time to treatment, inadequate collateral, and comorbidities further increase the likelihood of poor functional outcome after EVT [4]. While these factors are associated with post-procedural outcomes, identifying more practical prognostic indicators remains crucial for better prediction and management after EVT.

Arterial stiffness is assessed with noninvasive devices and correlates well with age and vascular risk factors [5]. Prior studies have repeatedly reported that arterial stiffness is related to poor prognosis in a variety of populations, including healthy individuals, the elderly, sedentary people, and patients with heart or kidney disease [6,7,8]. In acute ischemic stroke, patients with increased arterial stiffness are more likely to experience severe neurological deficits, poor functional outcomes, and major adverse cardiovascular events [9,10,11]. In a previous study, we reported a relationship between increased arterial stiffness and intracranial atherosclerosis [12]. Patients with intracranial atherosclerosis generally experience worse outcomes after EVT [13]. Therefore, an arterial stiffness assessment may predict an unfavorable prognosis after EVT. However, the relationship of arterial stiffness with post-EVT outcome has not been elucidated. In this study, we hypothesized that arterial stiffness is a predictor of poor functional outcome in patients with acute ischemic stroke treated with EVT.

## 2. Materials and Methods

### 2.1. Study Population

We retrospectively enrolled consecutive acute ischemic stroke patients treated with EVT at a single tertiary stroke center between 18 June 2020 and 29 November 2022. The study included patients with the following characteristics: (1) age 20 years or older; (2) underwent EVT for large vessel occlusion associated with neurologic symptoms found on angiographic evaluation such as computed tomography angiography, magnetic resonance angiography, or digital subtraction angiography; (3) pre-stroke modified Rankin Scale score ≤2; and (4) performed an arterial stiffness test. Exclusion criteria encompassed patients who did not undergo EVT, those in poor health and unable to undergo arterial stiffness measurement, those with a severe pre-existing disability, or those without confirmed reperfusion status from follow-up imaging 24 h after EVT. This study was conducted in strict adherence to the Declaration of Helsinki on Ethical Principles for Medical Research Involving Human Subjects. This study was also approved by the Institutional Review Board of Severance Hospital of the Yonsei University Health System, and informed consent was waived due to the retrospective nature of this study (approval number: 4-2024-0445).

### 2.2. Endovascular Treatment and Outcome

EVT was performed for eligible patients who had large vessel occlusion and met the criteria of the current guidelines [1]. Briefly, patients with a small infarct core and less than 6 h from the last known normal time were eligible. In cases with between 6 and 24 h from the last known normal time, patients who had salvageable brain tissue according to multimodal computed tomography or magnetic resonance imaging were candidates for EVT [14,15]. Patients with unclear onset were also eligible for EVT if they had sufficient mismatch volume on perfusion imaging. Intravenous tissue plasminogen activator was administered within 4.5 h of stroke onset, unless contraindicated. Mechanical devices were the primary choice for EVT, containing stent retrievers and/or direct aspiration. The devices were selected by target vessel site, degree of tortuosity, and interventionalist preference. Device pass number indicates the number of times the mechanical device was passed during the EVT procedure. Successful recanalization was evaluated on follow-up computed tomography angiography or magnetic resonance angiography at 24 h after EVT and defined as modified Thrombolysis in Cerebral Infarction grade 2b or 3. Poor functional outcome was defined as a modified Rankin Scale score ≥3 at 3 months.

### 2.3. Arterial Stiffness Assessment

Arterial stiffness was evaluated during hospitalization using brachial–ankle pulse wave velocity (baPWV) measured by an automated device (VP-1000 plus, Collins, Komaki, Japan). This device calculated two parameters: the distance traveled by the pulse wave (between the brachial artery in the upper arm and the posterior tibial artery in the ankle) and the time it took for the wave to travel that distance (latency). baPWV was then calculated by dividing the distance by the latency and expressed in meters per second. Measurements were obtained from both the right and left sides of the body, and the higher of the two baPWV values was used for further analysis.

### 2.4. Statistical Analysis

A chi-square test or Fisher’s exact test was used for categorical variables, and the independent t test or Mann–Whitney U test was used for continuous variables to investigate significant differences between groups. Receiver operating curve (ROC) analysis was conducted to calculate the optimal cutoff value, with the highest Youden index of baPWV predicting poor functional outcome. Univariable and multivariable logistic regression analyses were performed to observe significant and independent predictors of poor functional outcome. Covariates were age, sex, National Institutes of Health Stroke Scale (NIHSS) score, and variables with *p* < 0.05 in univariable analysis. Subgroup analyses were performed using multivariable logistic regression analysis to explore the prognostic effect of arterial stiffness according to clinical characteristics. A nomogram was established based on the multivariable logistic regression model, and the area under the curve (AUC) and calibration curve were plotted to confirm the predictive ability and validity of the nomogram. Net reclassification improvement (NRI) and integrated discrimination improvement (IDI) were further employed to evaluate the predictive performance of the nomogram compared to classic risk factors. The two-tailed *p* value for significance was less than 0.05. All statistical analyses were performed using IBM SPSS Statistics software Version 26.0 (IBM, Armonk, New York, NY, USA), SAS analytics software Version 9.4 (SAS Institute, Cary, NC, USA), and R software Version 4.3.2 (R Foundation for Statistical Computing, Vienna, Austria, http://www.r-project.org (accessed on 20 June 2024)).

## 3. Results

**Figure 1:**
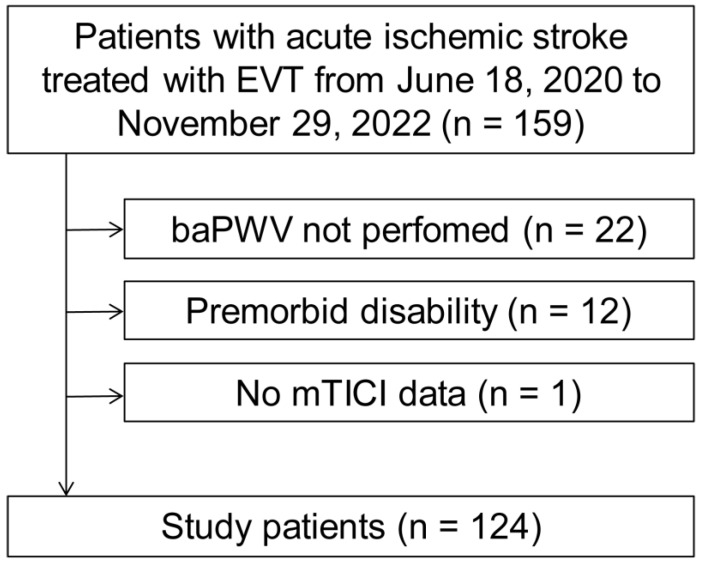
Patient flow chart. baPWV, brachial–ankle pulse wave velocity; EVT, endovascular treatment; mTICI, modified Thrombolysis in Cerebral Infarction.

During the study period, 159 patients were diagnosed with acute ischemic stroke and treated with EVT. Among them, patients who were not assessed for arterial stiffness (*n* = 22), presented with severe premorbid disability (*n* = 12), or had unknown reperfusion status (*n* = 1) were excluded. A total of 124 patients were finally included (Figure 1). The mean age of the study patients was 71.9 ± 11.8 years, and 57.3% were men. The median NIHSS score at admission was 12 (interquartile range, 7.0–16.8).

### 3.1. Factors Associated with Poor Functional Outcome

Poor functional outcome was present in 58 patients (46.8%) and was associated with older age, higher initial NIHSS score, hypertension, no atrial fibrillation, and lower high-density lipoprotein cholesterol (all *p* values < 0.05). Procedure-related factors such as device pass number and recanalization failure were linked to poor functional outcome (all *p* values < 0.05). The ROC analysis determined a baPWV cutoff value > 16.57 m/s with an AUC of 0.719, sensitivity of 93.1%, and specificity of 43.9% (Figure S1). Regarding the arterial stiffness measurement, both the continuous and cutoff values of baPWV were associated with poor functional outcome (all *p* values < 0.05) (Table 1).

### 3.2. Univariable and Multivariable Logistic Regression Analyses for Poor Functional Outcome

Univariable logistic regression analysis showed that both continuous and cutoff values of baPWV were significantly associated with poor functional outcome (all *p* values < 0.05). Older age, higher initial NIHSS score, hypertension, no atrial fibrillation, device pass number, recanalization failure, and time interval from admission to baPWV measurement were also associated with poor functional outcome (all *p* values < 0.05). In the multivariable logistic regression analysis, both continuous and cutoff values of baPWV were independently associated with poor functional outcome after adjusting for covariates (continuous value of baPWV: odds ratio [OR] 1.104, 95% confidence interval [CI] 1.007–1.211; baPWV > 16.57 m/s: OR 8.640, 95% CI 1.581–47.228) (Table 2).

### 3.3. Subgroup Analysis of Poor Functional Outcome

**Figure 2:**
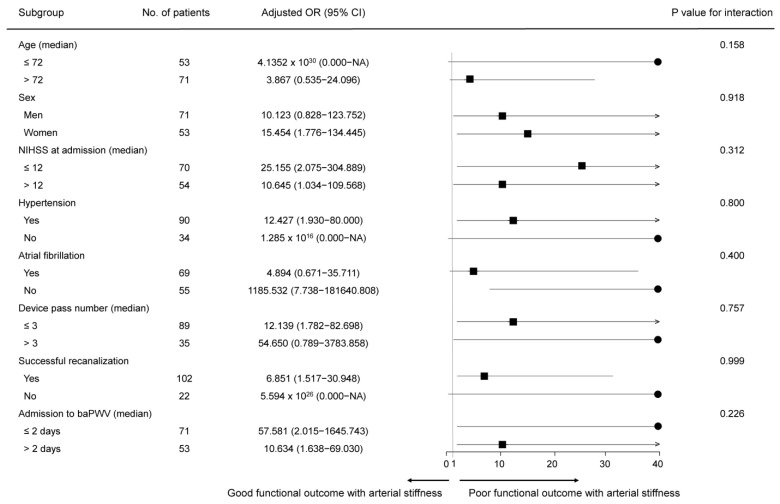
Subgroup analysis. baPWV, brachial–ankle pulse wave velocity; CI, confidence interval; NIHSS, National Institutes of Health Stroke Scale; OR, odds ratio.

Subgroup analyses were performed according to clinical characteristics of age, sex, initial NIHSS score, hypertension, atrial fibrillation, device pass number, successful recanalization, and time interval from admission to baPWV measurement. No significant interactions were observed in any subgroup with respect to poor functional outcome. Moreover, the point estimates across all strata increased with arterial stiffness (*p* values for interactions ≥ 0.05) (Figure 2).

### 3.4. Prognostic Model of Poor Functional Outcome

**Figure 3:**
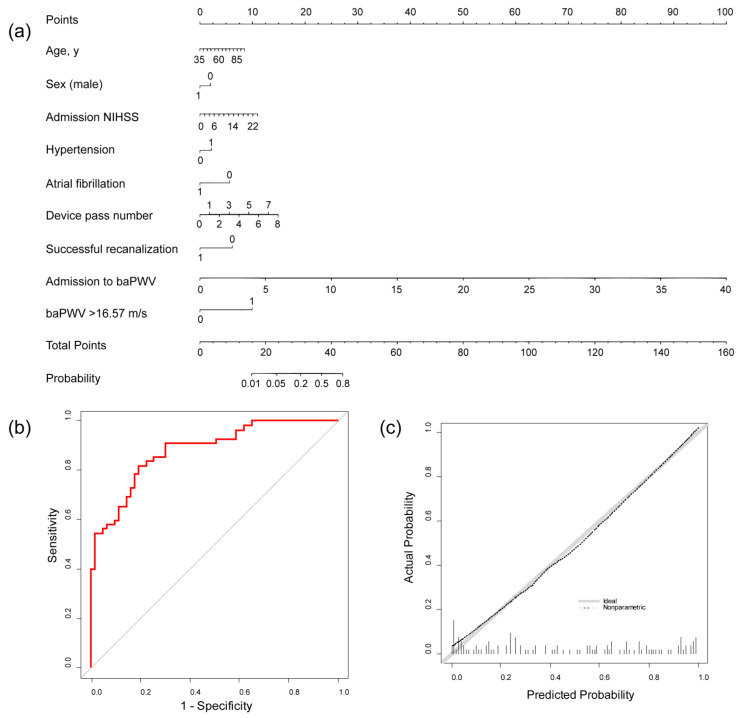
Nomogram for predicting poor functional outcome. (**a**) Development of the nomogram. For example, 1 point is awarded if the patient is 40 years old, 2 points if female, 3 points if admission NIHSS is 6, 0 points for no hypertension, 0 points for atrial fibrillation, 2 points if the device pass number is 1, 0 points for successful recanalization, 12 points if the time from admission to baPWV is five days, and 10 points if baPWV is above the cutoff. The total points for this patient were 30. Drawing a line on the nomogram directly down from this point, the probability of a poor functional outcome is 0.176. (**b**) ROC curve of the nomogram. (**c**) Calibration curve of the nomogram, baPWV, brachial–ankle pulse wave velocity; NIHSS, National Institutes of Health Stroke Scale; ROC, receiver operating curve.

A prognostic model of functional outcome at 3 months utilizing arterial stiffness was constructed as the nomogram based on the multivariable logistic regression analysis (Figure 3a). The predicted probability of poor functional outcome at 3 months was calculated using the following equation: predicted probability = 1/(1 + exp [−A]), where A = −6.063 + 0.031 × age − 0.442 × male + 0.099 × admission NIHSS + 0.474 × hypertension − 1.227 × atrial fibrillation + 0.402 × device pass number − 1.343 × successful recanalization + 0.54 × admission to baPWV + 2.156 × baPWV > 16.57 m/s. The predictive ability of the prognostic model showed an AUC of 0.882 (95% CI 0.822–0.941) (Figure 3b). The calibration curve showed a close approximation between the observed and predicted probabilities, indicating that the prognostic model is well calibrated (Figure 3c).

### 3.5. Predictive Performance of Prognostic Model

We designed three models that predict poor functional outcome at 3 months. Model 1 included baseline variables (age, sex, and NIHSS score), Model 2 included all covariates (age, sex, NIHSS score, hypertension, atrial fibrillation, device pass number, successful recanalization, and time interval from admission to baPWV measurement), and Model 3 added baPWV > 16.57 m/s to all covariates. The predictive performance of the models was compared using NRI and IDI. The nomogram model (Model 3) had an NRI of 1.083 (0.779–1.387) and an IDI of 0.363 (0.274–0.452) compared with Model 1 (*p* < 0.05), along with an NRI of 0.529 (0.186–0.873) and an IDI of 0.046 (0.009–0.083) in comparison with Model 2 (*p* < 0.05) (Table 3).

## 4. Discussion

This study demonstrated that arterial stiffness measured by baPWV was independently associated with poor functional outcome at 3 months in patients treated with EVT following acute ischemic stroke. Subgroup analysis indicated that the negative impact of arterial stiffness on post-EVT outcome persisted across various clinical characteristics. In addition, the nomogram was constructed using arterial stiffness and showed significant prognostic performance over traditional vascular risk factor models.

Previously, several predictors of poor outcome after EVT have been reported [3,4,16,17]. Regarding baseline characteristics, older age, higher initial NIHSS score, comorbidities such as diabetes and hypertension, systolic blood pressure, glucose and biomarkers, and late treatment are reported [3]. Imaging factors include large infarction, poor collaterals, proximal occlusion, and white matter hyperintensity [4,17]. Procedural factors include symptomatic intracranial hemorrhage, blood pressure variability, and blood pressure controls after recanalization [18,19]. However, these predictors are insufficient to predict outcome after EVT, and more practical and accurate predictors may be helpful for tailored treatment and risk stratification after EVT.

Arterial stiffness is readily evaluated with baPWV and is a well-established marker of ischemic stroke outcome. The stiffening of a large artery is influenced by aging and vascular risk factors and independently linked to stroke prognosis [5]. Association between arterial stiffness and poor functional outcome was also reported in acute ischemic stroke [10]. Prognostic impacts remained valid across stroke subtypes [20]. Arterial stiffness is also an independent determinant of all-cause and vascular mortality after acute ischemic stroke [21] and is involved in prothrombogenic conditions such as inflammation, physical inactivity, or muscle mass deficit [22,23,24]. In this regard, we hypothesized that arterial stiffness is relevant to poor functional outcome at 3 months after EVT in patients with acute ischemic stroke.

Our findings indicated that arterial stiffness may be an independent prognostic marker after EVT. We speculated on potential reasons for the adverse effect of arterial stiffness with the following hypothesis. First, arterial stiffness is characterized by the decreased elastic fiber of the aortic arteries and consequently reduces the buffering effect against blood pressure. Prior studies have shown the correlation between arterial stiffness and higher systolic blood pressure with greater variability [25]. It has been reported that blood pressure variability is a poor prognostic factor after EVT [26]. Second, our previous study found a relationship between increased arterial stiffness and intracranial atherosclerosis [12]. Patients with intracranial atherosclerosis generally experience poorer outcomes after EVT due to higher rates of recanalization failure, re-occlusion, procedural complications, and the need for repeat interventions [13,27]. Third, arterial stiffness was positively correlated with the initial NIHSS score (Table S1). Initial stroke severity is often associated with larger and more critical infarctions, which are associated with poor outcome [28]. Lastly, arterial stiffness was significantly associated with older age, hypertension, and large artery atherosclerosis (Table S2). Comorbidities are known to strain the body’s resources and reduce resilience to acute ischemic stroke [29]. Thus, these factors may limit stroke recovery and worsen functional outcome after EVT.

We developed a nomogram to predict the 3-month outcome after EVT by combining conventional risk factors with arterial stiffness. This prognostic model showed good predictive ability for poor functional outcome and was well calibrated. In addition, the nomogram performed better than baseline variable or covariate models in predicting poor functional outcome. Furthermore, because of the advantages of visualization tools over traditional risk stratification methods [29], the nomogram may be a feasible and user-friendly tool for predicting post-EVT outcome in clinical practice.

This study had several limitations. First, although patients were enrolled prospectively, the study data were evaluated retrospectively, which introduces potential confounders and selection bias. Second, some patients were excluded due to the lack of arterial stiffness data or a pre-existing disability, which may influence the results. Third, this study was conducted in a Korean population at a single institution; further studies with multicenter and multiethnic populations are needed to generalize the results. Fourth, our prognostic model was not validated using independent data. Future validation with external cohorts is needed to increase generalizability and ensure model robustness. Fifth, the noninvasive gold standard for arterial stiffness is carotid–femoral PWV, but previous studies have shown that baPWV is highly correlated with cfPWV and has the simplicity of testing and patient convenience [5].

## 5. Conclusions

We revealed that arterial stiffness in EVT patients is an independent predictor of poor functional outcome in addition to conventional risk factors. Measuring arterial stiffness is useful for predicting prognosis in acute ischemic stroke patients treated with EVT, as it is simple, requires little patient cooperation, and is relatively inexpensive.

## Figures and Tables

**Table 1:** Demographic and clinical characteristics.

	Total (*n* = 124)	Poor Functional Outcome		*p* Value
		Yes (*n* = 58)	No (*n* = 66)	
Age, y	71.9 ± 11.8	74.3 ± 11.6	69.8 ± 11.7	0.034
Sex (male)	71 (57.3)	32 (55.2)	39 (59.1)	0.660
Admission NIHSS	12.0 [7.0, 16.8]	13.0 [9.0, 18.3]	9.0 [6.0, 15.0]	0.005
Risk factors				
Hypertension	90 (72.6)	48 (82.8)	42 (63.6)	0.017
Diabetes	39 (31.5)	18 (31.0)	21 (31.8)	0.925
Dyslipidemia	44 (35.5)	17 (29.3)	27 (40.9)	0.178
Atrial fibrillation	69 (55.6)	26 (44.8)	43 (65.2)	0.023
Previous stroke	29 (23.4)	11 (19.0)	18 (27.3)	0.276
Current smoking	18 (14.5)	7 (12.1)	11 (16.7)	0.468
Peripheral artery disease	15 (12.1)	9 (15.5)	6 (9.1)	0.274
Blood tests, mg/dL				
Total cholesterol	148.0 [129.0, 176.0]	142.0 [120.8, 170.8]	160.0 [133.0, 184.5]	0.123
Triglyceride	93.0 [72.0, 125.0]	101.5 [78.0, 127.5]	87.0 [69.5, 125.0]	0.149
HDL-C	42.0 [35.0, 50.0]	39.5 [32.8, 47.5]	45.0 [38.0, 51.5]	0.008
LDL-C	86.0 [66.0, 111.0]	81.0 [65.5, 112.3]	96.0 [66.0, 111.0]	0.245
Stroke subtypes				
Cardioembolism	65 (52.4)	26 (44.8)	39 (59.1)	0.548
Large artery atherosclerosis	21 (16.9)	13 (22.4)	8 (12.1)	
Stroke of other determined etiology	5 (4.0)	3 (5.2)	2 (3.0)	
Incomplete evaluation	3 (2.4)	1 (1.7)	2 (1.6)	
Negative evaluation	11 (8.9)	6 (10.3)	5 (7.6)	
Two or more causes	19 (15.3)	9 (15.5)	10 (15.2)	
Endovascular procedure				
Device pass number	3.0 [2.0, 4.0]	3.0 [2.0, 4.0]	2.0 [1.0, 3.0]	0.001
Onset to puncture, min	362.0 [200.0, 722.0]	367.0 [237.5, 785.0]	358.0 [170.0, 530.3]	0.239
Onset to reperfusion, min	400.0 [222.0, 763.0]	400.0 [267.5, 812.0]	399.5 [192.8, 563.3]	0.167
Successful recanalization	102 (82.3)	43 (74.1)	59 (89.4)	0.026
Good collateral	88 (71.0)	39 (67.2)	49 (74.2)	0.391
Arterial stiffness measurement				
Heart rate, bpm	75.0 [63.3, 88.8]	78.0 [66.5, 91.3]	71.0 [62.5, 85.0]	0.065
Admission to baPWV, day	2.0 [1.0, 3.0]	3.0 [2.0, 4.0]	2.0 [1.0, 3.0]	<0.001
baPWV, m/s	20.12 [16.12, 23.33]	21.56 [18.72, 26.07]	18.55 [14.79, 21.23]	<0.001
baPWV > 16.57 m/s	91 (73.4)	54 (93.1)	37 (56.1)	<0.001

Continuous and categorical variables are shown as mean ± standard deviation or median (interquartile range) and number (%), respectively. Successful recanalization was mTICI 2b or 3. Good collateral was Tan scale > 1. baPWV, brachial–ankle pulse wave velocity; HDL-C, high-density lipoprotein cholesterol; LDL-C, low-density lipoprotein cholesterol; mTICI, modified Thrombolysis in Cerebral Infarction; NIHSS, National Institutes of Health Stroke Scale.

**Table 2:** Logistic regression analysis for poor functional outcome.

	Univariable		Multivariable ^a^	
	OR (95% CI)	*p* Value	OR (95% CI)	*p* Value
Age, y	1.035 (1.002–1.068)	0.037	1.031 (0.975–1.090)	0.280
Sex(male)	0.852 (0.418–1.739)	0.660	0.643 (0.217–1.902)	0.425
Admission NIHSS	1.093 (1.026–1.164)	0.006	1.104 (1.006–1.212)	0.037
Hypertension	2.743 (1.177–6.392)	0.019	1.607 (0.436–5.921)	0.476
Diabetes	0.964 (0.451–2.062)	0.925		
Dyslipidemia	0.599 (0.283–1.266)	0.180		
Atrial fibrillation	0.435 (0.211–0.896)	0.024	0.293 (0.096–0.893)	0.031
Previous stroke	0.624 (0.266–1.462)	0.278		
Current smoking	0.686 (0.247–1.906)	0.470		
Peripheral artery disease	1.837 (0.612–5.517)	0.279		
Stroke subtypes				
Cardioembolism	0.559 (0.254–1.234)	0.150		
Large artery atherosclerosis	1.558 (0.513–4.736)	0.434		
Others ^b^	Reference			
Device pass number	1.461 (1.130–1.889)	0.004	1.495 (1.064–2.100)	0.020
Onset to puncture, minute	1.001 (1.000–1.001)	0.237		
Onset to reperfusion, minute	1.001 (1.000–1.002)	0.162		
Successful recanalization	0.340 (0.128–0.906)	0.031	0.261 (0.059–1.158)	0.077
Good collateral	0.712 (0.327–1.550)	0.392		
Heart rate, bpm	1.011 (0.995–1.028)	0.183		
Admission to baPWV, day	1.804 (1.328–2.451)	<0.001	1.716 (1.193–2.468)	0.004
baPWV, m/s	1.126 (1.051–1.206)	0.001	1.104 (1.007–1.211)	0.035
baPWV > 16.57 m/s	10.581 (3.432–32.623)	<0.001	8.640 (1.581–47.228)	0.013

Successful recanalization was modified Thrombolysis in Cerebral Infarction of 2b or 3. Good collateral was Tan scale > 1. The adjusted ORs of the covariates were analyzed with the cutoff of baPWV entered. baPWV, brachial–ankle pulse wave velocity; CI, confidence interval; NIHSS, National Institutes of Health Stroke Scale; OR, odds ratio. ^a^ adjusted for age, sex, admission NIHSS, hypertension, atrial fibrillation, device pass number, successful recanalization, and admission to baPWV. ^b^ Stroke subtypes other than cardioembolism or large artery atherosclerosis.

**Table 3:** Predictive performance of nomogram.

	Predictive Ability (95% CI)	*p* Value
NRI		
vs. Model 1	1.083 (0.779–1.387)	<0.001
vs. Model 2	0.529 (0.186–0.873)	0.003
IDI		
vs. Model 1	0.363 (0.274–0.452)	<0.001
vs. Model 2	0.046 (0.009–0.083)	0.015

baPWV, brachial–ankle pulse wave velocity; CI, confidence interval; IDI, integrated discrimination improvement; NIHSS, National Institutes of Health Stroke Scale; NRI, net reclassification improvement. Model 1: age, sex, and NIHSS. Model 2: age, sex, NIHSS, hypertension, atrial fibrillation, device pass number, successful recanalization, and admission to baPWV. Nomogram (Model 3): Model 2 + baPWV > 16.57 m/s.

## Data Availability

The data that support the findings of this study are available from the corresponding author upon reasonable request.

## Supplementary Materials

The following supporting information can be downloaded at https://www.mdpi.com/article/10.3390/jcm13144198/s1, Figure S1: ROC analysis of the optimal cutoff value of baPWV to predict poor functional outcome; Table S1: Pearson and Spearman correlation matrix; Table S2: Demographic and clinical characteristics.
