# Supplementary material for: Arterial Stiffness Predicts the Outcome of Endovascular Treatment in Patients with Acute Ischemic Stroke
Source: J Clin Med. 2024 Jul 18;13(14):4198. doi: 10.3390/jcm13144198 (PMC11278379; doi:10.3390/jcm13144198)
Supplement: Supplementary file 1 [file jcm-13-04198-s001.zip › jcm-3092727-supplementary.pdf]

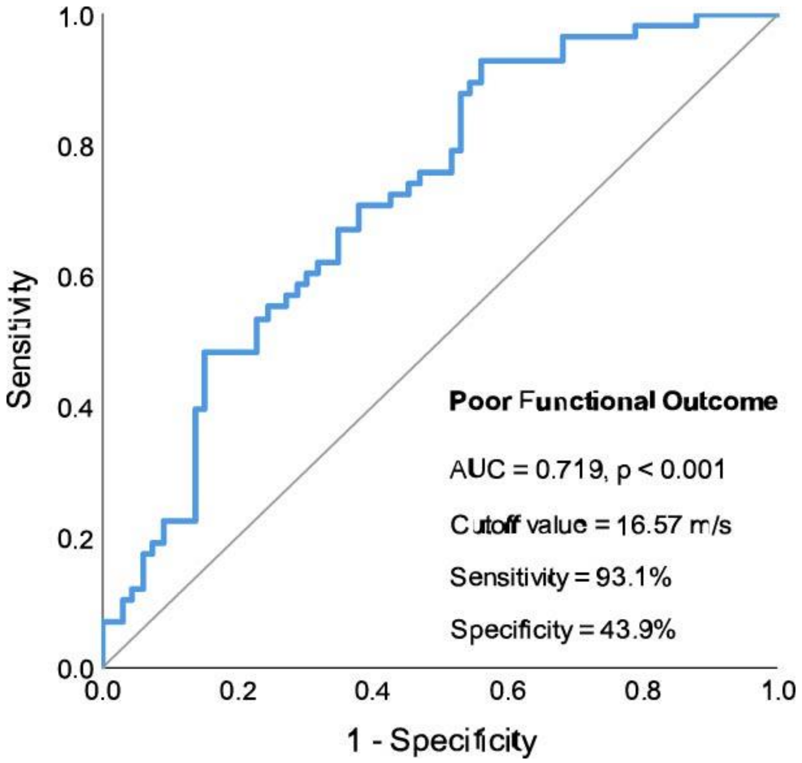

Figure S1. ROC analysis of the optimal cutoff value of baPWV to predict poor functional outcome. AUC, area under the curve; ROC, receiver operating curve.

Table S1. Pearson and Spearman correlation matrix.

Pearson correlation coefficient

Variable	Age	Admission NIHSS	Device pass number	Tan scale	baPWV
Age	1				
Admission NIHSS	0.163	1			
Device pass number	-0.055	0.056	1		
Tan scale	0.016	-0.183*	-0.077	1	
baPWV	0.366**	0.182*	-0.138	-0.057	1

Spearman correlation coefficient

Variable	Age	Admission NIHSS	Device pass number	Tan scale	baPWV
Age	1				
Admission NIHSS	0.165	1			
Device pass number	-0.068	0.052	1		
Tan scale	-0.015	-0.154	-0.117	1	
baPWV	0.487**	0.229*	-0.046	0.052	1

baPWV, brachial-ankle pulse wave velocity; NIHSS, National Institutes of Health Stroke Scale. *p<0.05. **P<0.01.

Table S2. Demographic and clinical characteristics.

	Arterial stiffness		p value
	Yes (n=91)	No (n=33)	
Age, y	75.3±9.9	62.6±11.8	<0.001
Sex (male)	57 (62.6)	14 (42.4)	0.044
Admission NIHSS	12.0 [7.0, 16.0]	10.5 [5.0, 17.2]	0.269
**Risk factors**			
Hypertension	77 (84.6)	13 (39.4)	<0.001
Diabetes	33 (36.3)	6 (18.2)	0.055
Dyslipidemia	30 (33.0)	14 (42.4)	0.331
Atrial fibrillation	47 (51.6)	22 (66.7)	0.137
Previous stroke	20 (22.0)	9 (27.3)	0.538
Current smoking	14 (15.4)	4 (12.1)	0.778
Peripheral artery disease	13 (14.3)	2 (6.1)	0.350
**Blood tests, mg/dL**			
Total cholesterol	137.5 [120.2, 164.8]	172.5 [146.5, 198.0]	0.001
Triglyceride	95.0 [72.0, 123.5]	91.5 [69.8, 139.8]	0.530
HDL-C	40.0 [33.0, 47.0]	46.5 [39.5, 55.0]	0.005
LDL-C	78.0 [63.0, 102.8]	103.5 [82.0, 136.3]	0.003
**Stroke subtypes**			
Cardioembolism	41 (45.1)	24 (72.7)	0.028
Large artery atherosclerosis	20 (22.0)	1 (3.0)	
Stroke of other determined etiology	3 (3.3)	2 (6.1)	

Incomplete evaluation	2 (2.2)	1 (3.0)	
Negative evaluation	9 (9.9)	2 (6.1)	
Two or more causes	16 (17.6)	3 (9.1)	
**Endovascular procedure**			
Device pass number	3.0 [2.0, 4.0]	3.0 [2.0, 4.0]	0.755
Onset to puncture, min	373.5 [208.0, 773.0]	358.0 [185.0, 524.8]	0.365
Onset to reperfusion, min	410.0 [232.5, 811.0]	399.5 [208.5, 561.5]	0.199
Successful recanalization	73 (80.2)	29 (87.9)	0.324
Good collateral	68 (74.7)	20 (60.6)	0.126

Continuous and categorical variables are shown as mean ± standard deviation or median (interquartile range) and number (%), respectively. Arterial stiffness was defined as baPWV >16.57 m/s. Successful recanalization was modified Thrombolysis in Cerebral Infarction 2b or 3. Good collateral was Tan scale >1. baPWV, brachial-ankle pulse wave velocity; HDL-C, high-density lipoprotein cholesterol; LDL-C, low-density lipoprotein cholesterol; NIHSS, National Institutes of Health Stroke Scale.
